# Human proximity suppresses fish recruitment by altering mangrove-associated odour cues

**DOI:** 10.1038/s41598-020-77722-7

**Published:** 2020-12-03

**Authors:** Rohan M. Brooker, Angelia L. Seyfferth, Alesia Hunter, Jennifer M. Sneed, Danielle L. Dixson, Mark E. Hay

**Affiliations:** 1 Centre for Integrative Ecology, School of Life and Environmental Sciences, Deakin University, Queenscliff, VIC 3225 Australia; 2 School of Marine Science and Policy, University of Delaware, Lewes, DE 19958 USA; 3 Department of Plant and Soil Sciences, University of Delaware, Newark, DE 19716 USA; 4 Smithsonian Marine Station at Fort Pierce, Fort Pierce, FL 34949 USA; 5 School of Biological Sciences and Aquatic Chemical Ecology Center, Georgia Institute of Technology, Atlanta, GA 30332-0230 USA

**Keywords:** Behavioural ecology, Environmental impact

## Abstract

Human-driven threats to coastal marine communities could potentially affect chemically mediated behaviours that have evolved to facilitate crucial ecological processes. Chemical cues and their importance remain inadequately understood in marine systems, but cues from coastal vegetation can provide sensory information guiding aquatic animals to key resources or habitats. In the tropics, mangroves are a ubiquitous component of healthy coastal ecosystems, associated with a range of habitats from river mouths to coral reefs. Because mangrove leaf litter is a predictable cue to coastal habitats, chemical information from mangrove leaves could provide a source of settlement cues for coastal fishes, drawing larvae towards shallow benthic habitats or inducing settlement. In choice assays, juvenile fishes from the Caribbean (Belize) and Indo-Pacific (Fiji) were attracted to cues from mangroves leaves and were more attracted to cues from mangroves distant from human settlement. In the field, experimental reefs supplemented with mangrove leaves grown away from humans attracted more fish recruits from a greater diversity of species than reefs supplemented with leaves grown near humans. Together, this suggests that human use of coastal areas alters natural chemical cues, negatively affecting the behavioural responses of larval fishes and potentially suppressing recruitment. Overall, our findings highlight the critical links that exist between marine and terrestrial habitats, and the importance of considering these in the broader conservation and management of coastal ecosystems.

## Introduction

Connectivity between emergent coastal vegetation and subtidal marine ecosystems can play a crucial environmental role, mediating productivity, community composition, and ecosystem functioning^1–3^. However, many coastal habitats, both above and below water, are rapidly changing due to anthropogenic pressure; becoming degraded, fragmented, and less biodiverse^4–6^, with the effects of these impacts often unclear^7,8^. Thus, identifying the ecological links between associated ecosystems is essential for effective conservation and resource management^9,10^. While human-driven changes to coastal landscapes are often clearly apparent, for instance the conversion of wild areas to agriculture or urban environments, the consequences of these actions for adjacent marine systems is generally less obvious. However, even small changes to land use can alter coastal marine environments; for instance, pollutants and nutrients in runoff that alter marine chemistry can affect the behaviour of marine animals^11–13^. Because ecological processes that can enhance or decrease coastal resilience are often behaviourally driven and mediated by chemosensory cues^14,15^, subtle changes to the chemosensory environment could appreciably affect ecosystem function and dynamics.

A diverse array of aquatic taxa has evolved acute chemosensory systems, with waterborne chemicals providing a rich source of environmental information^15^. These chemical cues can mediate a range of important behavioural processes, informing foraging patterns^16^, navigation^17^, predator–prey dynamics^18^, habitat selection^14^, and interspecies interactions^19^. For species with an initial planktonic life stage, such as most fishes and marine invertebrates, chemoreception can play an especially important role, helping to orient larvae and juveniles towards key habitats and facilitating recruitment processes^20^. Because chemical signals influence critical ecological processes, it is crucial that we know cue sources and consequences. Information cues often have a marine origin, such as the odour of corals, seaweeds, or conspecifics^14,21^. However, due to the intimate relationship between coastal plant communities and marine habitats, some marine species respond to cues from emergent, or even non-aquatic coastal vegetation^22,23^. How such cues may be affected by coastal development is inadequately understood. Because coastal plant communities are being lost, or altered, at alarming rates^24–26^, and produce chemical cues that are critical for coastal marine species and communities, understanding the sources of these cues and processes affected by them is both timely and crucial.

Along tropical and subtropical coastlines, mangrove forests comprise one of the most ubiquitous plant communities. These salt-tolerant plants represent globally important ecosystems, providing habitat for communities of terrestrial, estuarine, and marine organisms^27^, including the juvenile stages of aquatic species that migrate elsewhere as adults, such as to nearby coral reefs^28^. In addition to facilitating biodiversity, mangroves sequester carbon, provide coastal protection, build land, accumulate and assimilate pollutants, and stabilize water conditions^29^. Several aspects of mangrove ecology suggest they could provide recruitment cues for coastal fishes, including fish species associated with fringing and lagoonal coral reefs. The worldwide distribution of tropical mangroves largely overlaps that of reef building scleractinian corals^30^, with mangroves growing in oligotrophic areas with limited freshwater input often occurring alongside fringing coral reefs and associated habitats^28,31–33^. In addition, numerous ‘coral reef fishes’ are in fact, multi-habitat species, with the juveniles and adults of many also associated with mangroves^34^. Finally, while mangroves hold leaves year-round, they continuously drop some leaves with rates of litter fall often peaking in warmer, or wetter months^35,36^, overlapping with periods of high recruitment by reef fishes^37,38^. Thus, standing plants could produce cues to mark coastal systems, degraded leaves that sink could provide similar cues, and leaves drifting from shore could provide a trail of cues leading back to the shallow, structurally complex benthic environments essential for post-settlement survival of coastal fishes.

If this is the case, variations in the chemical composition of mangroves or their decomposing litter could alter the sensory information subsequently released. Globally, mangrove ecosystems are at high risk, reducing in area at rates equal to, or greater than, coral reefs and rainforests^25,39^. Documented mangrove losses over the last quarter of the twentieth century were consistently between 35–86%, with stands becoming smaller, more fragmented, and less biodiverse due to the combined effects of agriculture, aquaculture, tourism, urban development and overexploitation^25,40,41^. While recent analysis suggests that rates of loss have substantially slowed in the twenty-first century^42,43^, the future of mangrove ecosystems remains uncertain. Pollution is also a major cause of mangrove deterioration, with many of the chemicals present in sewage, runoff, and other direct inputs (e.g. nitrates, phosphates, and heavy metals) reducing mangrove growth and condition^44,45^. As mangroves naturally accumulate compounds from their environments, many pollutants are incorporated into the plant’s tissues^46^. These then have the potential to be subsequently remobilized when those tissues degrade, with greater bioavailability than those held in sediments^47^. The presence of pollutants will also alter the microbial community associated with the water column, sediment, and plant material^44,48^, which could further impact litter decomposition and associated chemical cues.

Given that environmental chemicals can influence the behaviour of marine larvae and that anthropogenic impacts to mangroves are likely altering marine chemistry, it is critical to determine if and how mangroves mediate juvenile recruitment, and if this role is compromised due to anthropogenic activities. To this end we examined, (1) if juvenile fishes are attracted to the odour of mangrove leaf litter, (2) how this varied among different leaf types, (3) if juvenile fishes distinguished between litter collected near versus remote from human settlements, and (4) if these behavioural responses reflected patterns of recruitment under field conditions.

## Results

### Do chemical cues from mangroves attract reef fishes?

**Figure 1:**
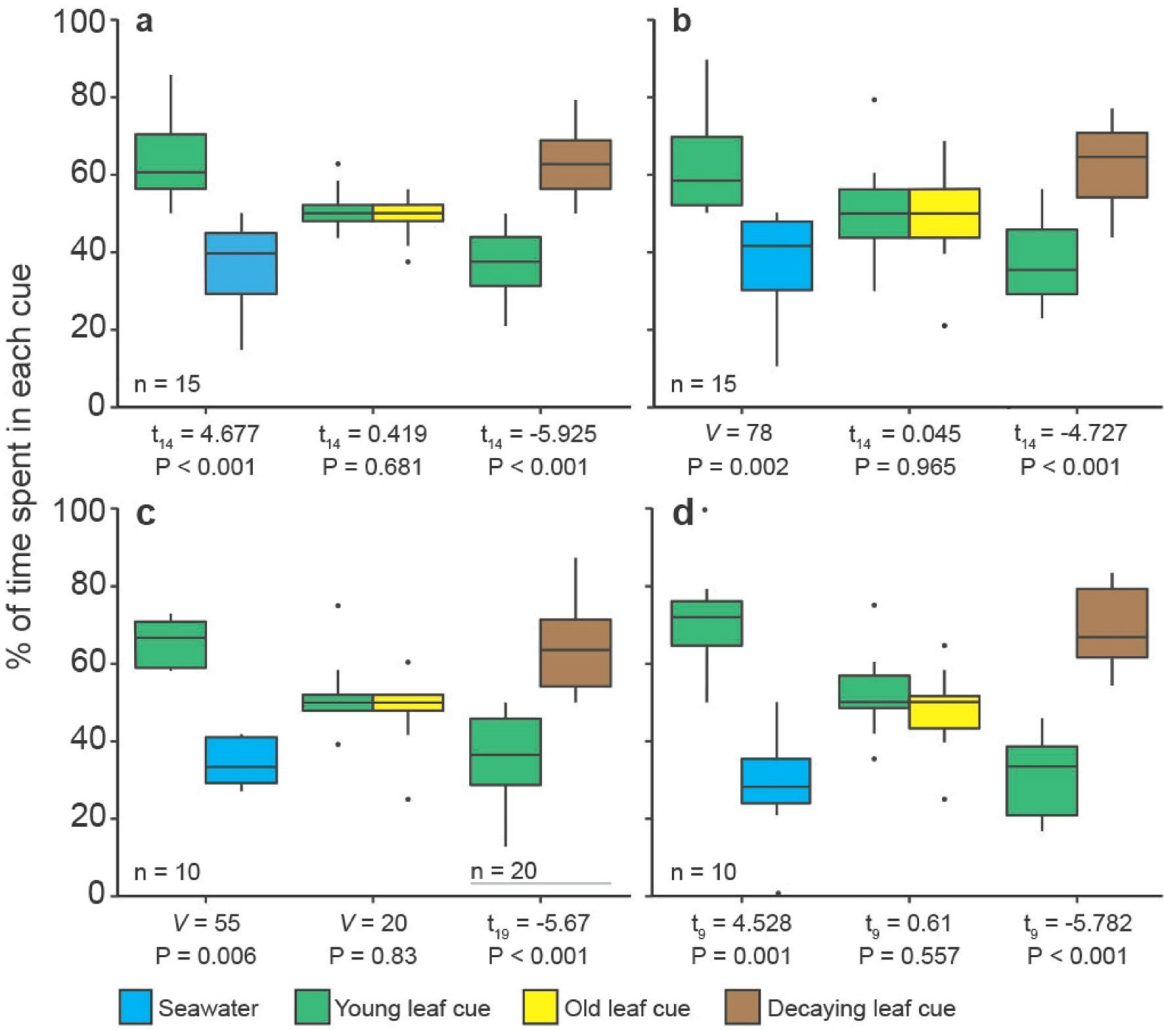
Results of paired-choice trials in Belize and Fiji testing the responses of two fish species in each region to the odour of mangroves leaves at different stages of growth and decay. Belizean species were (**a**) *Thalassoma bifasciatum*, (**b**) *Stegastes partitus*. Fijian species were (**c**) *Chromis viridis*, and (**d**) *Dascyllus reticulatus*. Three comparisons were conducted; either (i) young leaves vs. blank sea water, (ii) young leaves vs. old leaves from the plants, or (iii) young leaves vs. decaying leaves from beneath the plants. Boxplots show median values (horizontal lines), interquartile range (boxes), and minimum and maximum values (whiskers). *p* values are displayed below each comparison, calculated using either a paired-sample t-test or paired-sample Wilcoxon test. n = number of fish per comparison.

To assess whether different mangrove odours influenced the behaviour of juvenile reef fishes, and whether response patterns were generalizable between species or geographic locations, we conducted a series of paired-choice experiments in a two-channel choice flume. These experiments were conducted in both Fiji (South Pacific Ocean) and Belize (Western Atlantic Ocean), with two common reef-associated fish species used per location; *Chromis viridis* and *Dascyllus reticulatus* in Fiji and *Thalassoma bifasciatum* and *Stegastes partitus* in Belize. All four fishes exhibited similar responses to the odour of mangrove leaves (Fig. 1a–d). All four species preferred the odour of young leaves over blank seawater, no species distinguished between the odour of young green and old yellow leaves still on the tree, and all four preferred the odour of senescent, submerged leaves to young leaves collected from the tree.

**Figure 2:**
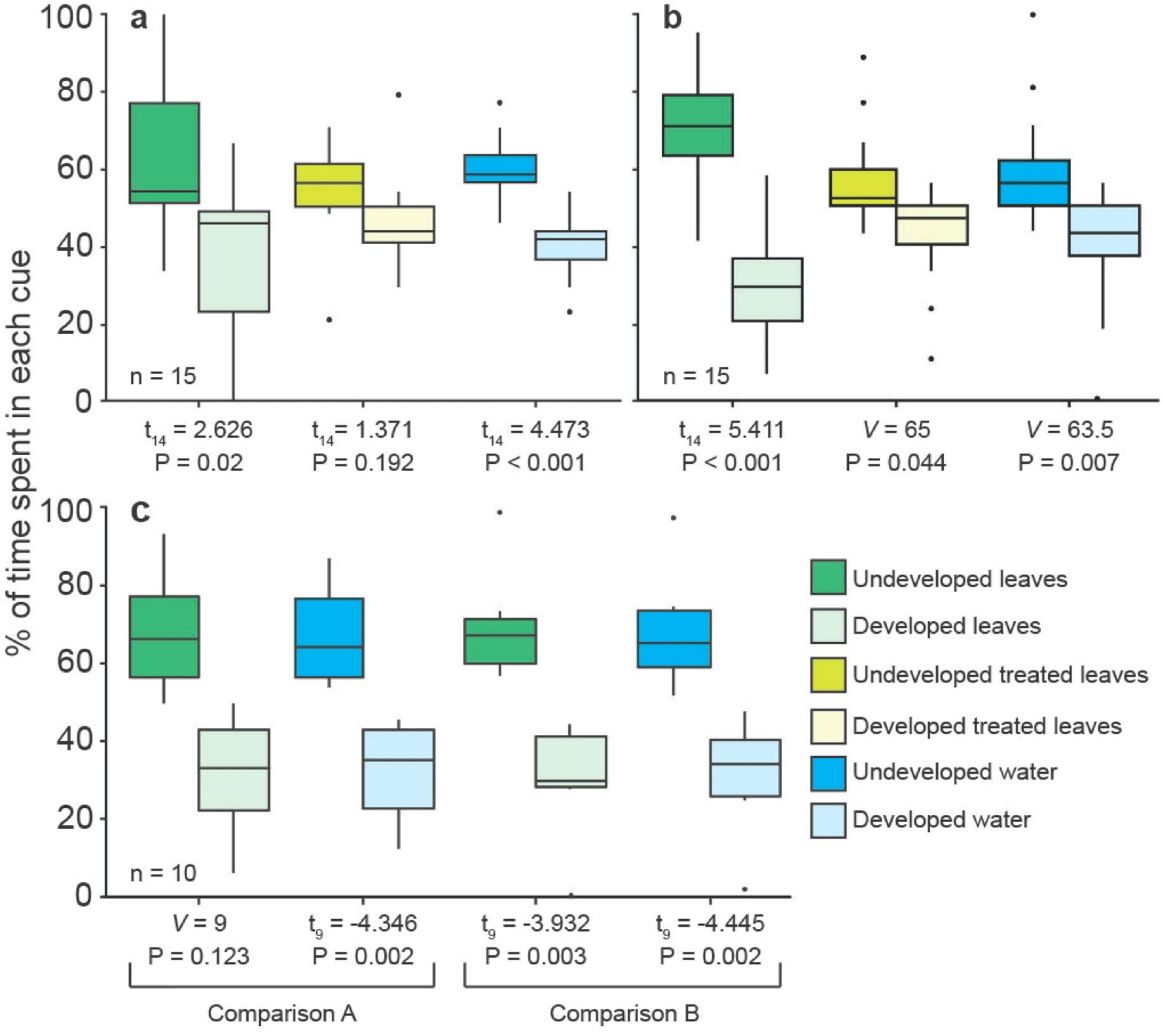
Results of paired-choice trials in Belize and Fiji testing the responses of fish species in each region to the odour of mangroves leaves or water collected from sites with human development vs limited human development. Belizean species were (**a**) *Thalassoma bifasciatum*, (**b**) *Stegastes partitus*. The sole Fijian species was *Dascyllus reticulatus* (**c**). For both Belizean species three comparisons were conducted; either (1) decaying leaves from an undeveloped site (Twin Cays) vs. decaying leaves from a developed site (South Water Cay), (2) decaying leaves from each site treated with NaOCl, and (3) water from each site. For *D. reticulatus,* Comparison A tested responses to either decaying leaves or water from an undeveloped site (Nukulau Island) or developed site (Suva). Comparison B tested responses to either decaying leaves or water from a second undeveloped site (Namuka) or developed site (Korovou). Boxplots and statistical procedures are as in Fig. 1.

When presented with senescent mangrove leaves from a site with human development versus senescent leaves from a site with limited human development both Belizean species preferred the odour of those from the undeveloped location (Fig. 2a, b). This preference was also seen towards water collected from each site. Interestingly, when leaves from both sites were treated to remove the associated microbial film the one labrid tested, *T. bifasciatum*, ceased to distinguish between odours; however, whether this is due to differences in the microbiome or is related to the treatment itself is not clear Similarly, the one Fijian species tested, *D. reticulatus,* preferred water from undeveloped locations in two separate comparisons; Suva (developed) versus Nukulau Island (undeveloped), and Korovou (developed) versus Namuka (undeveloped). This preference was extended to the odour of senescent leaves from Namuka versus those from Korovou, but not for leaves from Nukulau versus those from Suva (Fig. 2c).

### Do mangrove chemical cues influence settlement site selection?

**Figure 3:**
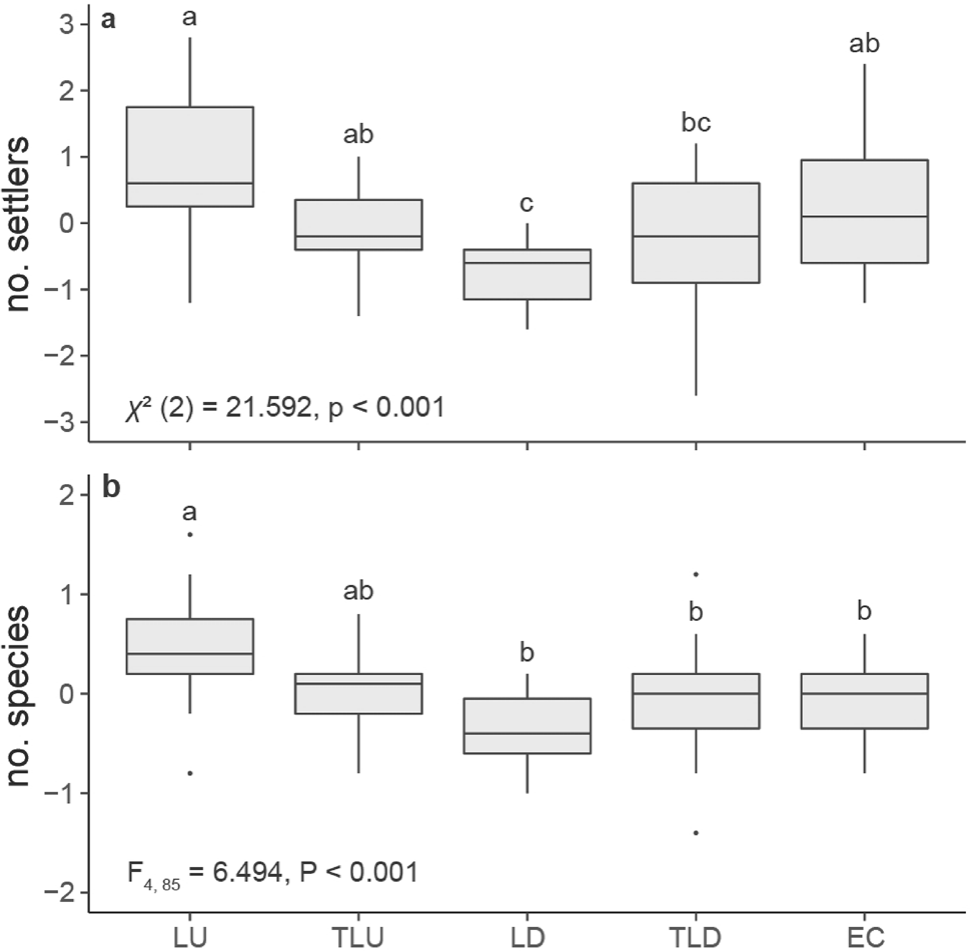
Results of a patch reef experiment testing whether the odour of mangrove leaves collected near human development (from South Water Cay) vs those from near limited human development (Twin Cays) influenced natural patterns of fish settlement in Belize. Patch reef treatments were: empty control (EC), leaves from the undeveloped site (LU), treated leaves from the undeveloped site (TLU), leaves from the developed site (LD), and treated leaves from the developed site (TLD). Values displayed are for total settlement or species number residualised with respect to even distribution within the replicate (*n* = 18 per treatment). Letters indicate significant differences as determined by post hoc multiple comparisons (*p* < 0.05; see Supplementary Tables S2, S3). Boxplots are as in Fig. 1.

To test whether the odour of decaying mangrove leaves from sites near human development vs those from sites with limited human development influenced natural patterns of fish settlement we conducted an experiment in Belize using constructed patch reefs that contained either NaOCl-treated or unmodified leaves from the developed or undeveloped location along with a control containing no leaves. NaOCl treatment reduced the microbial load on the leaves. Nine fish species settled onto the experimental patch reef sets: *Canthigaster rostrata, Gymnothorax moringa, Halichoeres bivittatus, Pomacanthus arcuatus,* a *Scarus* sp., *Stegastes adustus, Stegastes leucostictus, S. partitus, and T. bifasciatum*. However, *T. bifasciatum* was by far the most common settler accounting for 88.6% of fish recorded (101 fish out of 114). Treatment had a significant effect on settlement (Fig. 3), with both total settlement and the diversity of settlers significantly higher on patch reefs containing unmodified undeveloped leaves than those containing unmodified leaves from the developed location. Total settlement was also significantly higher on both the control reefs and those containing treated leaves from the undeveloped site than to reefs containing the unmodified leaves from developed sites. There was no difference in either variable between the treated leaves from the undeveloped and developed site. However, as in the paired choice experiment results above, it is possible the treatment itself had an effect.

## Discussion

Mangrove-associated chemical cues influenced the behaviour of multiple species of settlement-stage fishes in both the Caribbean and tropical Pacific with our findings suggesting that these cues are diminished or reversed by mangrove proximity to humans. This was true for mangroves growing adjacent to a large city (Suva, Fiji with a population of ~ 170,000), but also for those growing on South Water Cay, Belize (an island of ~ 0.06 km^2^ comprised primarily of tourist resorts). This suggests that even sparse human populations on isolated islands may suppress the effectiveness of chemical cues that fishes use to find and recruit to appropriate coastal habitats. Thus, humans not only remove adult fish and brood-stock by fishing but may also be indirectly suppressing the ability of local fish populations to recover via interference with the chemical cues that organisms use to identify suitable habitat choices during recruitment.

For coastal fishes, an ability to identify and orientate towards mangroves would be invaluable as mangrove roots, detritus, and associated benthic communities create structural complexity and habitat diversity where this may otherwise be limited, offering shelter from predators, increasing the abundance of food resources, and reducing competition. As the distribution of mangrove stands often overlaps that of corals and other sessile invertebrates within oligotrophic environments, the innate ability to recognize mangrove cues would likely prove valuable for fishes that associate with a range of shallow habitats, but especially those species that recruit to mangroves as juveniles and move to reefs as they mature^28,34,49^. Our choice experiments demonstrated that fish species associated with both reefs and rubble were attracted to mangrove odours, likely due to the close relationship between these habitats and mangroves at the study sites. Fishes were not simply responding to novel chemical cues; all four species distinguished between leaves at different stages of growth and decay, while two of the three tested distinguished between those collected near versus far from humans. Thus, the attraction for mangrove odour and the ability to distinguish nuances of this odour seems to have been selected for among different species from at least two families occupying different oceans. Recruiting reef species may use numerous odours^20^, as well as other sensory modes (e.g. vision and hearing^50^) to select recruitment sites; decaying plant material may act as a sensory ‘booster’ in tandem with other sensory cues, increasing the likelihood that larvae will identify appropriate habitats.

The similar behavioural responses observed between locations suggests that human activities have a generalized effect on recruiting fishes in that they can initiate an avoidance behaviour. In Fiji, our developed sampling locations were close to the capital city of Suva, within several miles of a fishing port, an industrial area, and dense urban development. In contrast, activities at the developed Belizean site of South Water Cay consisted primarily of tourist resorts and associated boat traffic. Our low-impact sites were more similar between locations, with little human activity occurring near collection sites. The similar behavioural responses observed highlight the need to identify the underlying causes of these negative chemical cues and determine the concentrations at which their presence has an adverse effect on fish behaviour.

Differences in the composition of the chemical cues produced as leaf litter breaks down could involve a number of pathways. For instance, chemicals may accumulate from the environment that are subsequently released as leaves decay or cues may differ due to environmental effects on leaf microbiomes. While the chemical composition of the leaves used in our behavioural experiments was not tested, there is some evidence that this can vary between sites occupied and unoccupied by humans. For instance, both Cu and Zn were 4–5 times higher in leaves subsequently obtained from the developed site, South Water Cay, compared to the undeveloped site, Twin Cays, Belize (Supplementary Table S1). Because the presence of toxic metals can directly impact the health of aquatic environments^51^, excess amounts of these or other pollutants in mangrove leaves from the developed site could have repelled fishes directly, or indirectly via effects on associated microbes. Treating leaves with NaOCl to reduce the microbial load on the leaves had a variable effect on both behaviour and settlement in Belize. While it is possible that the treatment itself had some effect, in behavioural assays the labrid, *Thalassoma bifasciatum* ceased to differentiate between leaves from each site while the pomacentrid, *Stegastes partitus,* continued to prefer leaves from the undeveloped site. The variable responses between species suggests that, while broad trends are consistent, subtle differences in how species or families perceive and respond to these cues exist. In the field, both total settlement and settler species richness did not differ between NaOCl-treated leaves from sites with versus without humans but did differ between these sites for untreated leaves. The increased settlement on artificial patch reefs in Belize that contained NaOCl-treated leaves from the developed site compared to untreated leaves from the developed site suggests that microbes associated with the developed site may be deterring fish recruitment, although the treatment itself may have also had an effect. Little is known about the relationship between microbial communities and fish recruitment, however, it is well established that bacterial biofilms and the chemicals they produce act as settlement cues for many invertebrate larvae (see^52^), and in some cases both juvenile fishes and coral larvae respond similarly to chemical cues^14^. Analysis of microbiomes from decaying leaves from each site in Belize found 20 OTUs in significantly higher relative abundances on leaves from the developed site (*p* < 0.001, Supplementary Fig. S1). These include sulfate-reducing taxa as well as copiotrophic taxa that are often associated with shifts in marine microbial communities exposed to anthropogenic nutrient enrichment^53^.

That mangrove-associated chemicals provide recruitment cues for fishes and that these cues are modified by even modest-scale human occupation of nearby sites highlight the importance of incorporating the impacts of terrestrial landscapes into marine spatial planning and management, and indicate the value of implementing management strategies that limit the amount or type of chemicals entering the marine environment via terrestrial sources. Additional work of value would include determining the importance of these cues relative to other biological and hydrodynamic processes as well as the chemicals to which the juvenile fishes are responding. In addition, future work should examine the role of mangrove odours and related chemical cues on the settlement and behaviour of mangrove specialist species, in particular those of ecological or economic importance. However, that we found behavioural responses to mangrove leaves in species not tightly associated with mangroves highlights that mangroves provide critical cues and habitats for a range of juvenile fishes and that their continuing losses affect not only mangrove systems, but also adjacent communities such as coral reefs.

## Methods

### Study sites and species

Portions of this study were conducted within the Western Atlantic (Belize) and portions within the South Pacific (Fiji). In Fiji, work was conducted in January–February 2015, while most work in Belize occurred in June 2015 with some additional sampling (for leaf chemical analysis, see Supplementary Materials) in March 2016. In Belize, laboratory and field-based work was conducted at the Smithsonian's Research Station at Carrie Bow Cay (16° 48′ 9.8316″ N, 88° 4′ 54.8148″ W) using fishes from Carrie Bow Cay and plant material collected from neighbouring islands, while in Fiji, laboratory-based experiments were conducted using fishes and plant material collected from reefs and islands offshore from Labasa, Vanua Levu (16° 23′ 08.5″ S, 179° 19′ 52.5″ E) and surrounding the capital city of Suva, Viti Levu (18° 9′ 1.8432″ S, 178° 27′ 13.392″ E). Laboratory-based behavioural work focused on two common fish species associated with fringing reefs at each location; the bluehead wrasse, *Thalassoma bifasciatum*, and bicolor damselfish, *Stegastes partitus*, in Belize, and the blue-green chromis, *Chromis viridis*, and two stripe damselfish, *Dascyllus reticulatus*, in Fiji. In each location, these species were selected due to the high number of recruits present during the study period. These species are common in reef and non-reef habitats near mangrove forests and associate with microhabitats such as corals and other reef invertebrates^34^.

### Do chemical cues from mangroves attract reef fishes?

To assess the effects of odours from mangroves on reef fishes, we conducted choice assays using a two-channel flume (13 cm length × 4 cm width)^54^ in which individual fish were presented with two parallel water flows, each containing different chemical cues. In the flume, each water mass remained separated on either side of the main chamber without producing turbulence or eddies. Water was gravity fed into the flume at equivalent volumes (100 ml min^−1^) from both sources, with dye tests conducted to confirm that the two water sources continued to exhibit parallel water flow. Recently settled fish (1.5–2 cm total length) were collected by hand from reefs not associated with mangroves using nets and clove oil and held in aerated tanks until experimentation. Trials occurred within 6–24 h of collection with all fish used observed actively swimming and interacting with their environment prior to each trial. All replicates used unique individual fish and none were used in multiple assays. For each trial, a fish was placed into the centre of the flume at the downstream end. Following a two-minute habituation period, the position of the fish (left or right side of chamber) was recorded at five-second intervals for a period of two-minutes. The source of water to each side of the chamber was then reversed and the chamber was allowed to flush for a one-minute period. Then, a second two-minute habituation and two-minute test period were conducted. This controlled for any side preference fish may be exhibiting within the flume. Due to logistical constraints, the tester was aware of the cues being tested. For each test, significant differences in time spent in each water source were determined using either paired-sample t-tests, or Wilcoxon signed-rank tests if data did not met the assumption of normality. All analyses of paired-choice data were conducted using R^55^. Fish that remained on one side during the habituation and test periods were considered to not be exhibiting normal exploratory behaviour and so were excluded from the subsequent analysis (Supplementary Table S5)^56^.

To assess whether fishes were attracted to odours from mangroves, we soaked 15 leaves (~ 10 cm L from tip to base × 6 cm width at widest point of blade) in 20 L of untreated seawater for a period of 2 h; and tested fish behavioural responses to this water versus the same water but without the leaves. In Belize, all water was taken from the Carrie Bow Cay seawater system (the intake for this water is located approximately 25 m seaward from the island); in Fiji, all baseline water was from collections made at least 1 km from any reef or land. For this, and all subsequent tests, treatment water was used within 4 h of production to limit deterioration of odour molecules. In both Belize and Fiji, *Rhizophora* spp. mangroves were used to produce leaf chemical cues. Mangroves from this circumtropical genus were dominant at all sites, forming large coastal and offshore stands. In Belize, the genus is represented by *Rhizophora mangle*^57^, while in Fiji, it is represented by *R. stylosa*, *R. samoensis,* and a hybrid of both, *R. x selala*^58^. In Fiji, plants were only distinguished to genus due to their overlapping distributions and morphological and genetic similarity.

After determining that mangrove cues were attractive to all four species of reef fish tested, we also evaluated whether fishes were differentially attracted to (1) young (green) versus old (yellow) leaves collected while still on the plants, and (2) young leaves from the plants versus submerged, decaying leaves that had fallen from the plants and were collected as leaf litter at a depth of ~ 50 cm below standing plants. Latex gloves were worn during collection to reduce altering leaf microbial communities. In Belize, all fishes for these trials were collected from the Carrie Bow Cay reef, with leaves collected from the neighbouring Twin Cays (16° 49′ 43.2″ N, 88° 06′ 14.1″ W). In Fiji, fishes were either collected off shore from Labasa (*C. viridis*, young versus submerged leaves) or from fringing reef to the east of Suva (18° 08′ 45.7″ S, 178° 22′ 45.5″ E) (*C. viridis*, young versus older leaves and young leaves versus blank seawater; *D. reticulatus*, all trials). Leaves were either collected from offshore stands near Labasa or stands inshore from the Suva fringing reef.

We also assessed whether attraction to odours from senescent, submerged mangrove leaves differed depending on whether these leaves were collected beneath mangroves near human settlements or beneath mangroves remote from human settlements. Sites near versus remote from human settlements were determined based on proximity to anthropogenic development. In Fiji, developed sites were mangrove stands near central Suva city (18° 9′ 22.432″ S, 178° 26′ 47.468″ E) and the village of Korovou (18° 7′ 12.55″ S, 178° 25′ 51.269″ E). Undeveloped sites were: Nukulau Island (18° 10′ 27.632″ S, 178° 31′ 5.646″ E) and Namuka (18° 08′ 06.2 "S, 178° 21′ 22.8″ E). In Belize, the largely uninhabited Twin Cays was the undeveloped site while the neighbouring, and more populated, South Water Cay was the developed site (16° 49′ 43.2″ N, 88° 06′ 14.1″ W). These islands were approximately 2 km apart at their shortest distance. In addition to testing responses to leaves, we also tested the responses of fishes towards water collected at each of the leaf collection sites. Water was collected in 15 L containers and used within 4 h of collection. Water from each site did not differ in clarity or colour based on visual inspection. Fishes for comparison one (Suva vs Nukulau) were collected from the fringing reef near Makuluva Island (18° 11′ 17.9″ S, 178° 30′ 57.3″ E), while fishes for comparison two (Korovou vs Namuka) were collected from fringing reef to the east of Suva as above.

### Do mangrove chemical cues influence settlement site selection?

At Carrie Bow Cay, three groups of five patch reefs (i.e., 15 in all) were built in a large, sandy area 40 m from shore and 100 m from the nearest reef, with each group of five patch reefs comprising one replicate block of treatments. For each block, the five patch reefs were arranged in a circle with each patch reef 1.5 m from its adjacent patch reefs. Each block was separated from the other two blocks by ≥ 15 m. Each patch reef was approximately 40 cm in diameter and consisted of equal parts coral rubble and live *Acropora prolifera* coral surrounding a stimulus emission device (SED)^59^. SEDs were rectangular plastic containers (L = 15 cm, W = 10, H = 10) with opaque mesh sides and top that allowed dispersal of the odour of the mangrove leaf litter inside. Materials used to make the reefs obscured the SEDs from view. The five treatments included; a control containing an empty SED (empty control = EC), unmodified mangrove leaves from the undeveloped site (leaves unmodified = LU), unmodified mangrove leaves from the developed site (LD), mangrove leaves from the undeveloped site treated to reduce the microbial biofilm (TLU), and mangrove leaves from the developed site treated to reduce the microbial biofilm (TLD). Treatment to remove the original leaf-associated microbiome consisted of spraying leaves with the bactericidal agent sodium hypochlorite (NaOCl) diluted to 0.005% with deionized water until saturated and holding these in a sterile container for 10-min. Leaves were then flushed with seawater until no chemical odour could be detected. The order and position of treatments within each patch reef block was randomised between each trial. This experiment was repeated for six consecutive nights during June 2015 yielding n = 18 per treatment (i.e. three replicates per treatment per night for six nights). Patch reefs were built each afternoon prior to sunset. The following morning at 0630 h, all fish recruits on each patch reef were identified and counted. All fish were removed from each patch reef and released onto nearby reef areas. To identify difference in settlement site selection while accounting for spatial and temporal variation in recruitment, total settlement and the total number of settler species to each patch reef was converted to a replicate residual for each block of treatments. This was done by dividing total settlement or species for all patch reefs in a block by five to give the expected numbers of settlers or species per reef if distributed evenly. Residual settlement and species for each patch reef equalled the actual minus expected settlement and species. As settlement data did not meet parametric assumptions, a Kruskal–Wallis rank sum test was used to determine whether residual settlement varied between treatments, with Dunn’s tests of multiple comparisons used for post hoc analysis. For species data, a one-way ANOVA was used with post hoc analysis conducted using Tukey’s HSD tests. All analyses of patch reef data were conducted using R^55^.

### Potential drivers of the behavioural patterns observed

Following the behavioural components of this study, we collected leaves from each site and analysed these for differences in elemental composition and in the community composition of their microbiomes. Because leaves for chemical analysis were not collected synchronous with our behavioural assays and because leaves for both components were from only one developed and one undeveloped site (Twin Cay and South Water Cay in Belize), we view these as preliminary data indicating possible drivers of the behavioural patterns we observed. Results from these efforts are presented in the Supplementary Materials.

### Ethics approval

All work using animals was approved by the Georgia Institute of Technology and University of Delaware ethics committees and followed relevant guidelines and regulations.

## Supplementary information

Supplementary Informations.

## Data Availability

All data and code used to conduct the analysis in this manuscript is available at: 10.5281/zenodo.4284108.
