# Supplementary material for: Human proximity suppresses fish recruitment by altering mangrove-associated odour cues
Source: Sci Rep. 2020 Dec 3;10:21091. doi: 10.1038/s41598-020-77722-7 (PMC7713406; doi:10.1038/s41598-020-77722-7)
Supplement: Supplementary file 1 — Supplementary Informations. [file 41598_2020_77722_MOESM1_ESM.docx]

**Human proximity suppresses fish recruitment by altering mangrove-associated odour cues.**

**Supplementary information**

Rohan M. Brooker, Angelia L. Seyfferth, Alesia Hunter, Jennifer M. Sneed, Danielle L. Dixson, Mark E. Hay

**Corresponding author:** Rohan Brooker; rohan.m.brooker@gmail.com

**This file includes:**

Supplementary text

Figures S1-S2

Tables S1-S5

SI References

**Supplementary text**

Analysis of mangrove leaves from a developed and undeveloped site

To provide a preliminary assessment of what underlying factors might be driving the behavioural patterns seen in our paired-choice and patch reef experiments, supplementary analyses were conducted to identify whether the chemical composition or associated microbial communities of mangrove leaves differed between the developed and undeveloped study sites in Belize.

**Materials and Methods**

*(a) Chemical analysis of mangrove leaves*

To determine if living leaves at each site were concentrating different elements due to environmental differences, leaves were collected by hand from the developed (South Water Cay) and undeveloped site (Twin Cays) in Belize in March 2016. Nitrile gloves were worn at all times during collection. At both sites, sets of 20 green leaves were removed from 15 randomly selected *Rhizophora mangle* trees growing at the water’s edge. Leaves for each tree were placed into an individual zip lock bag and brought to Carrie Bow Cay Research Station. All leaves were thoroughly washed using distilled water and placed into individually marked paper bags. These were then placed into a drying oven set to 105**°** C where they remained for 96 hours to ensure they were thoroughly dried. Packages of dried leaves were then transferred to the University of Delaware, Newark, Delaware, for analysis.

To investigate differences in nutrients and metals between sites, all dried leaves for each sample were pulverized in a stainless-steel grinder and subject to various analyses. For elemental analysis, 0.2 g leaf tissue was subject to closed vessel microwave-assisted digestion (CEM MARS Xpress 6) with 7 mL of concentrated trace-metal grade nitric acid, diluted to 2% nitric acid with 18 MΩ water and analysed with inductively coupled plasma-mass spectrometry (ICP-MS, Agilent 7500) (1). Total concentrations were determined for Cr, Cu, Zn, As, Cd, Pb, Be, Al, Ti, V, Mn, Fe, Co, Ni, Se, Rb, Sr, Mo, Ag, Sb, Cs, Ba, U. Leaf tissue was also subject to 2% acetic acid extraction, and an analysis of nitrate-N (NO3-N), ammonium-N (NH_4_-N), and phosphate-P (PO_4_-P) was conducted using an AutoAnalyzer (Bran & Luebbe AA3). Quality control was assured by quantifying samples against matrix-matched standards and by including blanks and standard checks every 10 samples. Differences in the mean of each metal(loid) or nutrient in leaf samples from each site (n=15) were determined using two-sample t-tests, or Wilcoxon rank sum tests if data did not met the assumptions of normality or homogeneity of variance. All analyses were conducted using R (2).

*(b) Microbial analysis of mangrove leaves from the developed and undeveloped site*

Because submerged, senescent leaves were most attractive to fishes and this differed between developed and undeveloped sites, we set out to assess differences in the microbiomes of submerged leaves from each site in Belize. In addition, we compared the microbiomes of submerged leaves used in the patch reef experiment to test whether microbiomes differed between untreated and NaOCl-treated leaves from each site. All sampling occurred in June 2015. For the site comparison, submerged, decaying leaves were collected by hand from both the developed (South Water Cay) and undeveloped site (Twin Cays) in Belize. For the patch reef comparison, leaves for each treatment (unmodified leaves from the undeveloped site, NaOCl-treated leaves from the undeveloped site, unmodified leaves from the developed site, and NaOCl-treated leaves from the treated site) were removed from a randomly selected SED following the completion of trials. Nitrile gloves were worn at all times during collection. Collected leaves were placed in sterile plastic bags (Whirl-Pak) and returned to CBC for biofilm sampling. Mangrove leaves were swabbed vigorously on both sides with a sterile swab soaked in RNAlater. Each swab was placed in 1 ml RNAlater in a sterile microcentrifuge tube, transferred to the Smithsonian Marine Station, Fort Pierce, Florida, and stored at -80**°**C.

Prior to DNA isolation, samples were thawed and shaken in a TissueLyser II (Qiagen) at 20 oscillations/s for 2 minutes. Swabs were removed and bacteria remaining in RNAlater were pelleted by centrifugation at 10,000 rcm for 5 minutes. RNAlater was removed from bacterial pellets and DNA was isolated using the MoBio PowerSoil DNA Isolation Kit following the manufacturer’s protocol. 16S rDNA was amplified following the Earth Microbiome Project protocol (3) using barcoded primers 515fB (AATGATACGGCGACCACCGAGATCTACACGCTXXXXXXXXXXXXTATGGTAATTGTGTGYCAGCMGCCGCGGTAA) and 806r (CAAGCAGAAGACGGCATACGAGAT AGTCAGTCAG CC GGACTACNVGGGTWTCTAAT) (4). Amplification of each sample was performed in triplicate in a single step 35 cycle PCR using 5 PRIME HotMaster Mix starting with an initial denaturation step (3 min at 94°C), followed by 35 cycles (45 sec at 94 °C, 60 sec at 50 °C, 90 sec at 72°C), and a final elongation step (10 min at 72°C). Triplicates were combined and visualized via gel electrophoresis. Amplicon products were quantified using a Qubit fluorometer with a Qubit dsDNA BR Assay Kit, pooled (240 ng/sample) and purified using QIAquick PCR Purification Kit (Qiagen).

The cleaned amplicon pool was submitted to the Smithsonian Institution’s Laboratory for Analytical Biology for paired-end sequencing on the MiSeq platform using the MiSeq V2 300-cycle Reagent Kit. The sequence data were processed using QIIME (5). Paired ends were joined with a maximum of 40% difference within the region of overlap. Barcodes and primers were removed, and sequences were quality filtered. The median read length of the remaining sequences was 253bp. Sequences were clustered at 97% identity using the subsampled open-reference operational taxonomic unit (OTU) picking method (6) without removal of singletons. OTUs were picked and taxonomically classified with uclust (7) using the curated Greengenes reference database (8). OTUs that were classified as mitochondria or chloroplasts were removed from analysis. Microbial community composition was compared among treatments by analysis of similarity (ANOSIM; 999 permutations) and non-metric multidimensional scaling (NMDS) based on the weighted unifrac measure of distance in QIIME. Differential abundance analyses were performed in QIIME using the DESeq2 package (8, 9). All sequences have been submitted to the NCBI SRA database under the BioProject ID PRJNA630764.

**Results**

*(a) Chemical analysis of mangrove leaves from the developed and undeveloped site*

There were significantly higher contents of some nutrients and metal(loid)s in leaves collected from the developed site than from the undeveloped site (Table S1). Of the metal(loid) and nutrient contents measured that were above the detection limit, significant differences in Cu, Zn, As, Ba, Sr, Fe, Ti, PO_4_-P and NH_4_-N were observed between sites. Notably, Cu and Zn were 4x and ~5x higher, respectively, in leaves from the developed site than the undeveloped site (Table S1). While leaf tissue concentrations for As were also significantly higher by ~3.5x for the developed site than the undeveloped site, the values were low (above the detection limit but below the minimum reporting limit for As). Leaf tissue concentrations of Mn and Rb and were above the detection limit, but no differences were observed between sites. Leaf tissue concentrations of NO3-N and Cr, Cd, Pb, Be, Al, V, Co, Ni, Se, Mo, Ag, Sb, Cs, Ba, U were below the detection limit and thus are not shown.

*(b) Microbial analysis of mangrove leaves from a developed and undeveloped site*

Bacterial biofilms on leaves from the undeveloped and developed sites did not differ significantly in overall composition (R = -0.074, p = 0.621). However, 34 OTUs were found to be differentially abundant on mangrove leaves from the developed and undeveloped sites (p < 0.001, Fig. S1). Leaves from the developed site had higher abundances of OTUs belonging to the phylum Deltaproteobacteria including sulfate-reducing Desulfovibrionaceae and Desulfobulbaceae families as well several Gammaproteobacteria.

Microbial communities on leaves from the four patch reef treatments differed significantly from one another (ANOSIM, R = 0.444, p = 0.04, Fig S2). There were no significant pairwise comparisons among the four treatments according to p values; however, large R statistics suggest differences between each pair of treatments (Table S4) except for unmodified developed vs. unmodified undeveloped (R= -0.074, p = 0.713). The most pronounced taxonomical difference among these treatments was a high relative abundance of Deltaproteobacteria in the unmodified developed treatment (25.11%) compared to 9.37% in the unmodified undeveloped, 5.07% in the treated undeveloped, and 5.03% in the treated developed samples.


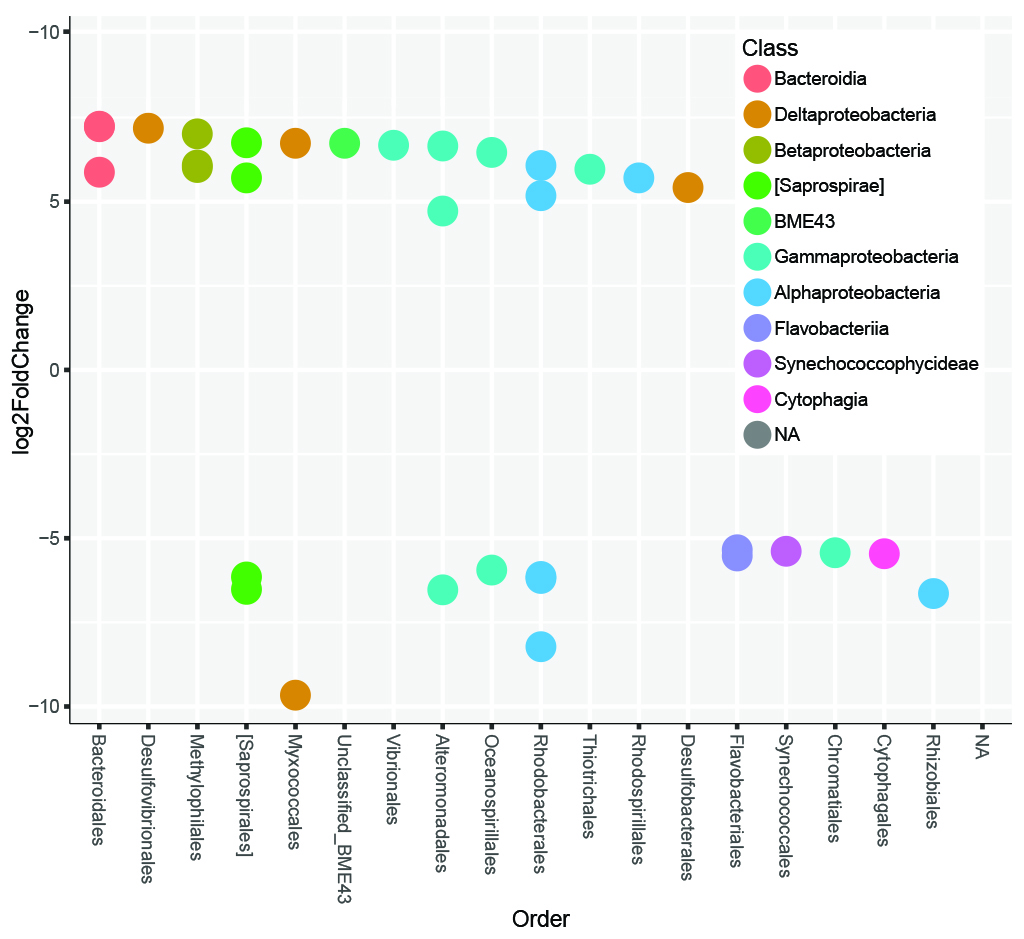


**Fig. S1.** Differentially abundant operational taxonomic units (OTUs) between bacterial biofilm communities sampled from the surfaces of mangrove leaves collected near developed and undeveloped sites in Belize. On the y-axis, point 0 represents the bacterial community present on leaves from the undeveloped site. Each coloured circle represents an OTU that is significantly more (log2FoldChange > 0) or less (log2FoldChange < 0) abundant on leaves from the developed site compared to leaves from the undeveloped site (i.e. point 0). Multiple circles of the same colour indicates that multiple OTUs from within the same taxonomic order were significantly different. N = 3 samples per site, p <0.001.


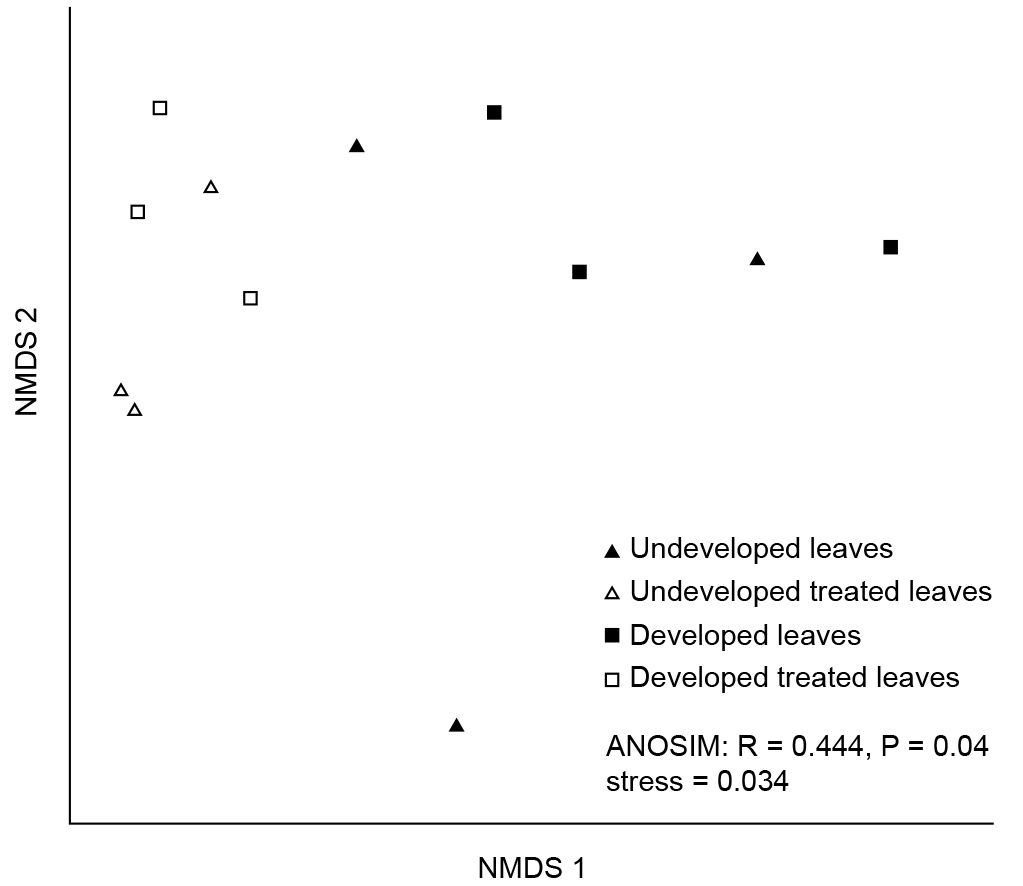


**Fig. S2.** Non-metric multidimensional scaling (NMDS) plot of bacterial biofilm communities sampled from the surfaces of mangrove leaves after removal from patch reefs based on the weighted unifrac distance. N per treatment = 3. Mangrove leaves were collected from Twin Cays (undeveloped site) or South Water Cay (developed site) in Belize. Leaves were either left as is or treated with NaOCl before addition to patch reef experiments.

**Table S1.** Elemental concentrations of mangrove leaves collected from the developed South Water Cay (n = 15) and undeveloped Twin Cays (n = 15). P-values indicate where the mean µg/g of an element differed between sampling sites, based on either a two-sample t-test (*t_28_*) or Wilcoxon test (W). Significant differences are in bold.

| Element | South Water Cay | | Twin Cays | | *t_28_* | *W* | *P-value* |
| --- | --- | --- | --- | --- | --- | --- | --- |
|  | Mean µg/g | SE | Mean µg/g | SE |  |  |  |
| As | 0.08 | 0.006 | 0.023 | 0.006 | 6.745 | - | **<0.001** |
| Ba | 0.35 | 0.086 | 0.116 | 0.116 | - | 168 | **0.007** |
| Ti | 0.928 | 0.064 | 0.399 | 0.037 | 7.118 | - | **<0.001** |
| Rb | 1.832 | 0.114 | 1.575 | 0.076 | 1.877 | - | 0.071 |
| Zn | 2.698 | 0.489 | 0.465 | 0.116 | *-* | 203 | **<0.001** |
| Mn | 3.13 | 0.72 | 2.379 | 0.668 | 0.765 | - | 0.451 |
| Cu | 4.544 | 0.267 | 0.967 | 0.695 | 4.803 | - | **<0.001** |
| Fe | 62.784 | 3.41 | 23.117 | 2.401 | 9.512 | - | **<0.001** |
| Sr | 171.156 | 8.235 | 114.508 | 5.29 | 5.788 | **-** | **<0.001** |
| NH_4_-N | 594.467 | 64.665 | 296.659 | 43.519 | 3.821 | **-** | **0.001** |
| PO_4_-P | 1278.867 | 91.679 | 911.367 | 45.813 |  | 196 | **<0.001** |

**Table S2.** Output from Dunn’s test of multiple comparisons of residualised settlement from patch reef experiment. Patch reef treatments were: empty control (EC), undeveloped site leaves (LU), undeveloped site leaves treated with NaOCl (TLU), developed site leaves (LD) and developed site leaves treated with NaOCl (TLD). n per replicate = 18. Significant differences are in bold.

| Comparison | Z | *P-value* |
| --- | --- | --- |
| TLD-EC | 0.88 | 0.473 |
| LD-EC | 2.928 | **0.017** |
| TLU-EC | 0.454 | 0.722 |
| LU-EC | -1.622 | 0.15 |
| LD-TLD | -2.048 | 0.068 |
| TLU-TLD | -0.426 | 0.67 |
| LU-TLD | 2.503 | **0.041** |
| TLU-LD | -2.474 | **0.033** |
| LU-LD | -4.551 | **<0.001** |
| LU-TLU | 2.077 | 0.076 |

**Table S3.** Output from Tukey-HSD test for residualised species from patch reef experiment. Patch reef treatments were: empty control (EC), undeveloped site leaves (LU), undeveloped site leaves treated with NaOCl (TLU), developed site leaves (LD) and developed site leaves treated with NaOCl (TLD). n per replicate = 18. Significant differences are in bold.

| Comparison | Diff | Lwr | Upr | *P-value* |
| --- | --- | --- | --- | --- |
| TLD-EC | < 0.001 | -0.436 | 0.436 | 1.000 |
| LD-EC | -0.278 | -0.713 | 0.158 | 0.394 |
| TLU-EC | 0.111 | -0.325 | 0.547 | 0.953 |
| LU-EC | 0.5 | 0.064 | 0.936 | **0.016** |
| LD-TLD | 0.278 | 0.158 | 0.713 | 0.394 |
| TLU-TLD | 0.111 | -0.325 | 0.547 | 0.953 |
| LU-TLD | -0.5 | -0.936 | -0.064 | **0.016** |
| TLU-LD | 0.389 | -0.047 | 0.825 | 0.103 |
| LU-LD | 0.778 | 0.342 | 1.213 | **<0.001** |
| LU-TLU | -0.389 | -0.825 | 0.047 | 0.103 |

**Table S4.** Pairwise comparisons of bacterial communities associated with mangrove leaves used in patch reef experiments following one-way ANOSIM. *R-values* are given above the diagonal in italics and *P-values* beneath the diagonal. LD = developed site leaves, TLD = treated developed site leaves, LU = undeveloped site leaves, TLU = treated undeveloped site leaves. n per treatment = 3.

|  | | *R-values* |  |  |  |
| --- | --- | --- | --- | --- | --- |
|  |  | LD | TLD | LU | TLU |
| *P-values* | LD | - | 0.666 | -0.074 | 0.814 |
|  | TLD | 0.106 | - | 0.370 | 0.370 |
|  | LU | 0.713 | 0.218 | - | 0.407 |
|  | TLU | 0.115 | 0.204 | 0.171 | - |

**Table S5**. Number of non-responsive individuals removed from each pairwise choice assay. Species are *Chromis viridis*, *Dascyllus aruanus*, *Thalassoma bifasciatum*, and *Stegastes partitus*.

| *Site* | *Species* | *Cue 1* | *Cue 2* | *No. removed* |
| --- | --- | --- | --- | --- |
| Fiji | *C. viridis* | Fresh leaves | Seawater | 0 |
|  |  | Fresh leaves | Old leaves | 1 |
|  |  | Fresh leaves | Decaying leaves | 1 |
|  | *D. aruanus* | Fresh leaves | Seawater | 1 |
|  |  | Fresh leaves | Old leaves | 2 |
|  |  | Fresh leaves | Decaying leaves | 0 |
|  |  | Suva water | Nukulau water | 0 |
|  |  | Suva leaves | Nukulau water | 0 |
|  |  | Korovou water | Korovou water | 0 |
|  |  | Korovou leaves | Korovou water | 0 |
| Belize | *T. bifasciatum* | Fresh leaves | Seawater | 1 |
|  |  | Fresh leaves | Old leaves | 2 |
|  |  | Fresh leaves | Decaying leaves | 0 |
|  |  | South Water water | Twin Cays water | 0 |
|  |  | South Water leaves | Twin Cays leaves | 1 |
|  |  | South Water treated leaves | Twin Cays treated leaves | 4 |
|  | *S. partitus* | Fresh leaves | Seawater | 2 |
|  |  | Fresh leaves | Old leaves | 6 |
|  |  | Fresh leaves | Decaying leaves | 3 |
|  |  | South Water water | Twin Cays water | 1 |
|  |  | South Water leaves | Twin Cays leaves | 1 |
|  |  | South Water treated leaves | Twin Cays treated leaves | 3 |

**Supplementary references**

1. A. L. Seyfferth, S. Fendorf, Silicate Mineral Impacts on the Uptake and Storage of Arsenic and Plant Nutrients in Rice (Oryza sativa L.). *Environ. Sci. Technol.* 46, 13176–13183 (2012).

2. R Core Team. R: a language and environment for statistical computing. R Foundation for Statistical Computing, Vienna, Austria. (2020).

3. J. G. Caporaso, C. L. Lauber, W. A. Walters, D. Berg-Lyons, J. Huntley, N. Fierer, S. M. Owens, J. Betley, L. Fraser, M. Bauer, N. Gormley, J. A. Gilbert, G. Smith, R. Knight, Ultra-high-throughput microbial community analysis on the Illumina HiSeq and MiSeq platforms. *Isme J.* 6, 1621-1624 (2012).

4. A. Apprill, S. McNally, R. Parsons, L. Weber, Minor revision to V4 region SSU rRNA 806R gene primer greatly increases detection of SAR11 bacterioplankton . *Aquat. Microb. Ecol.* 75, 129–137 (2015).

5. J. G. Caporaso, J. Kuczynski, J. Stombaugh, K. Bittinger, F. D. Bushman, E. K. Costello, N. Fierer, A. G. Peña, J. K. Goodrich, J. I. Gordon, G. A. Huttley, S. T. Kelley, D. Knights, J. E. Koenig, R. E. Ley, C. A. Lozupone, D. McDonald, B. D. Muegge, M. Pirrung, J. Reeder, J. R. Sevinsky, P. J. Turnbaugh, W. A. Walters, J. Widmann, T. Yatsunenko, J. Zaneveld, R. Knight, QIIME allows analysis of high-throughput community sequencing data. *Nat. Methods* 7, 335-336 (2010).

6. J. R. Rideout, Y. He, J. A. Navas-Molina, W. A. Walters, L. K. Ursell, S. M. Gibbons, J. Chase, D. McDonald, A. Gonzalez, A. Robbins-Pianka, J. Clemente, J. A. Gilbert, S. M. Huse, H. W. Zhou, R. Knight, J. G. Caporaso, Subsampled open-reference clustering creates consistent, comprehensive OTU definitions and scales to billions of sequences. *PeerJ* 2, e545 (2014).

7. R. C. Edgar, Search and clustering orders of magnitude faster than BLAST. *Bioinformatics* 26, 2460–2461 (2010).

8. T. Z. DeSantis, P. Hugenholtz, N. Larsen, M. Rojas, E. L. Brodie, K. Keller, T. Huber, D. Dalevi, P. Hu, G. L. Andersen, Greengenes, a Chimera-Checked 16S rRNA Gene Database and Workbench Compatible with ARB. *Appl. Environ. Microbiol.* 72, 5069–5072 (2006).

9. M. I. Love, W. Huber, S. Anders, Moderated estimation of fold change and dispersion for RNA-seq data with DESeq2. *Genome Biol.* 15, 550 (2014).
